# Three-dimensional anatomy and dorsoventral asymmetry of the mature *Marchantia polymorpha* meristem develops from a symmetrical gemma meristem

**DOI:** 10.1242/dev.204349

**Published:** 2024-11-29

**Authors:** Victoria Spencer, Eva-Sophie Wallner, Katharina Jandrasits, Natalie Edelbacher, Magdalena Mosiolek, Liam Dolan

**Affiliations:** Gregor Mendel Institute, Dr-Bohr-Gasse 3, 1030 Vienna, Austria

**Keywords:** Meristems, *Marchantia polymorpha*, Stem cell, Apical cell, Gemmae, Dorsoventrality

## Abstract

Meristems are three-dimensional (3D) generative structures that contain stem cells and produce new organs and tissues. Meristems develop in all land plants; however we know little about the spatial and temporal regulation of meristem structure in lineages such as bryophytes. Here, we describe the 3D meristem anatomy during the development of the liverwort *Marchantia polymorpha.* We show that the apical stem cell of the mature meristem is sub-apical, ventral, and in the outer cell layer. Mature meristem anatomy is therefore asymmetrical in the dorsoventral axis, which is reflected by the domain-specific protein localisation of Class III and Class IV Homeodomain-Leucine-Zippers (MpC3HDZ and MpC4HDZ), and by the promoter activity of Mp*YUCCA2*. The dorsoventral asymmetry that defines the mature meristem is absent in the juvenile meristems of asexual propagules known as gemmae. We discovered that anatomical dorsoventral asymmetry of the meristem forms after 1 to 2 days of gemmaling growth, and is accompanied by expression of the dorsal identity reporter MpC3HDZ. We conclude that the gemma meristem has arrested development and undergoes anatomical rearrangement to develop the 3D meristem structure of the mature plant.

## Teaser

**Summary:** The anatomy, symmetry and gene expression of the *Marchantia polymorpha* meristem changes during its lifespan, providing a foundational framework for identifying previously unreported mechanisms of meristem regulation.

## INTRODUCTION

The indeterminate growth of land plants is dependent on the maintenance of generative centres known as meristems. Meristems are three-dimensional (3D) structures that contain stem cells and their immediate daughter cells (collectively termed the stem cell niche), as well as an undifferentiated, proliferative zone that rapidly divides to produce the tissues of the plant body (Arnoux-Courseaux and Coudert, 2024; Eshed Williams, 2021). The anatomy and molecular regulation of flowering plant meristems are well described. Both the shoot apical meristem and root apical meristem are dome shaped and produce organs such as leaves and lateral roots behind the stem cell niche. Meristem anatomy is maintained in a steady state by the spatial restriction of gene expression to discrete domains (Kitagawa and Jackson, 2019; Yamoune et al., 2021). This steady state is established *de novo* during meristem formation in the embryo and is modified during developmental transitions such as reproduction (Barton, 2010; Bertran et al., 2024). Meristem architecture is therefore dynamically regulated through the life cycle of a plant.

All land plants produce meristems; however, their meristem anatomy is highly diverse (Arnoux-Courseaux and Coudert, 2024). Bryophytes have a haploid dominant phase (known as the gametophyte), and it is hypothesised that these meristems evolved independently to the meristems of the diploid dominant phase of flowering plants (the sporophyte) (Fouracre and Harrison, 2022). Flowering plants and gymnosperms typically have a multicellular stem cell population, whilst gametophyte bryophyte meristems often contain one or two stem cells known as apical cells. For example, the moss *Physcomitrium patens* has a 2D filamentous growth stage with a single stem cell at the apex of the filament. During the transition to 3D shoot growth, a tetrahedral apical cell with three cutting faces is generated, which gives rise to a spiral pattern of leaf-like phyllid initials (Harrison et al., 2009). The shape and number of cutting faces of the apical cell varies in different bryophyte lineages (Shimamura, 2016). The apical cell of leafy liverworts also typically has three cutting faces; the apical cell of simple thalloid liverworts is usually lenticular with two cutting faces; and the apical cell of complex thalloid liverworts, such as the Marchantiales, is typically wedge shaped with four cutting faces (Douin, 1925; Leitgeb, 1881; Shimamura, 2016). It is unclear how the spatio-temporal regulation of meristem architecture in each land plant lineage compares.

Molecular regulators of haploid meristem maintenance have been identified in bryophytes such as the liverwort *Marchantia polymorpha*. For example, CLAVATA LRR-RLK receptors and their CLAVATA3/Embryo surrounding region-related (CLE) peptide ligands are conserved in *M. polymorpha* (Hirakawa et al., 2020; Takahashi et al., 2021). However, MpCLV1-MpCLE2 signalling promotes stem cell proliferation in the *M. polymorpha* meristem, in contrast to CLAVATA1/2-CLV3 signalling in flowering plants, which inhibits stem cell proliferation (Hirakawa, 2022; Hirakawa et al., 2020). Other identified regulators include Mp*JINGASA* (Mp*JIN*) (Takahashi et al., 2023), Mp*PLETHORA* (Mp*PLT*) (Fu et al., 2024), Mp*AINTEGUMENTA* (Mp*ANT*) (Liu et al., 2024) and Mp*TDIF RECEPTOR* (Mp*TDR*) (Hirakawa et al., 2019). These studies suggest that some of the same genes control meristem function in *M. polymorpha* and flowering plants.

Our understanding of the genetic regulation of the *Marchantia* meristem has progressed significantly in recent years. However, current expression data in *M. polymorpha* meristems lack cellular resolution in three dimensions and focus primarily on the early development of young meristems in vegetative propagules known as gemmae. To understand the spatio-temporal regulation of the networks that control *M. polymorpha* meristem development, a 3D framework of meristem anatomy is needed. Here, we describe the cellular organisation of the *M. polymorpha* meristem, and we show that the anatomy and gene expression is distinct in each plane, and is dorsoventrally polarised. Inactive gemmae lack dorsoventral asymmetry, which becomes progressively established during the first 3 days of gemmaling growth. We conclude that the mature gemma meristem is arrested and undergoes substantial anatomical development to produce the steady-state meristem of the mature plant.

## RESULTS

### The anatomy of the mature *M. polymorpha* meristem is bilaterally symmetrical

The *M. polymorpha* thallus is flat, grows horizontally along the ground and bifurcates when grown in white light. Growth and morphogenesis occur at the tips of the thallus in notches between two lobes of differentiated tissues. In the notch, an apical cell acts as a self-renewing stem cell, which, together with its immediate daughter cells, constitute the stem cell niche. This stem cell niche produces undifferentiated proliferating cells that form the surface and internal tissues of the thallus. Collectively, the stem cell niche and the zone of undifferentiated proliferating cells constitute the meristem (Arnoux-Courseaux and Coudert, 2024; Kohchi et al., 2021; Shimamura, 2016).

**Fig. 1.**
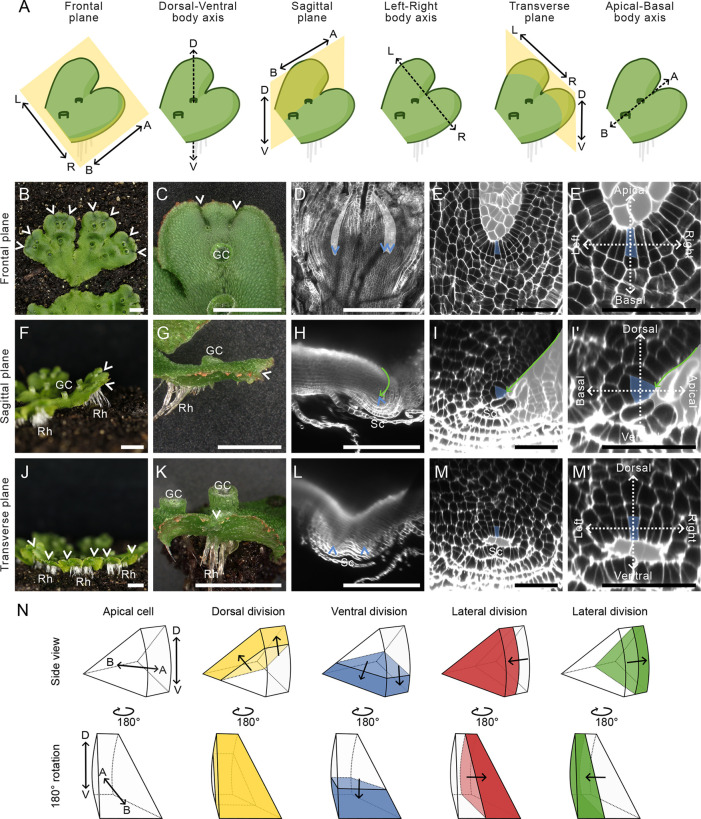
**The anatomy of the mature *M. polymorpha* meristem is bilaterally symmetrical**. (A) Schematic representation of the anatomical planes and body axes of the mature Tak-1 *Marchantia polymorpha* meristem. The frontal plane separates the dorsal (D) and ventral (V) sides; the sagittal plane separates the left (L) and right (R) sides; the transverse plane separates the apical (A) and basal (B) sides of the plant body. The meristem is positioned where the three planes intersect. (B-E′) Frontal views of the *M. polymorpha* plant. (B) The bifurcating plant body grows horizontally along the substrate. Arrowheads indicate notches. (C) Meristems are located at the tip of the plant in notches (white arrowheads). Gemma cups (GCs) are visible on the thallus surface. (D) Confocal microscopy image showing the stem cell niche (blue arrowheads) within the notch. (E,E′) Confocal microscopy image showing that the predicted apical cell (blue) is located at the base of the notch in the outer cell layer. (E′) A higher magnification of E. The apical-basal and left-right body axes are marked. (F-I′) Sagittal views of the *M. polymorpha* plant. (F) Rhizoids (Rh) grow from the ventral surface and GCs protrude upwards from the dorsal surface of the thallus. Arrowheads indicate notches. (G) Meristems are located at the tip of the thallus, but the notch (white arrowhead) is not visible in this orientation. (H) A light-sheet microscopy image showing the stem cell niche (blue arrowhead) at the apex of the thallus (green arrow). The stem cell niche is surrounded by scales (Sc). (I,I′) Confocal microscopy images showing that the predicted apical cell (blue) is positioned sub-apically and on the ventral side of the thallus apex. Green arrow indicates the thallus apex. (I′) A higher magnification of I. The dorsoventral and apical-basal body axes are marked. (J-M′) Transverse views of the *M. polymorpha* plant. (J) Rhizoids (Rh) form from the ventral surface. The heart-shaped notches (white arrowheads) are evident due to the slight upwards bending of the thallus. (K) The meristem is in the base of the notch (white arrowhead) and is flanked by the thallus lobes. GCs protrude from the dorsal thallus surface behind the notch. (L) Light-sheet microscopy image showing the stem cell niche (blue arrowheads) on the ventral surface of the thallus, surrounded by scales. (M,M′) Confocal microscopy images showing the ventrally localised predicted apical cell (blue). (M′) A higher magnification of M. The dorsoventral and left-right body axes are marked. In all confocal images, the space surrounding the meristem is marked in pale grey for clarity. (N) The stem cells in the meristem are wedge shaped in three dimensions. The stem cells divide in four directions to produce daughter cells. Together, the stem cells and their immediate daughter cells form the stem cell niche. Scale bars: 5* *mm in B,C,F,G,J,K; 500 µm in D,H,L; 50 µm in E,E′,I,I′,M,M′.

To define the 3D cellular architecture of the mature meristem, wild-type plants were grown from gemmae for 4 weeks in white light before fixation and clearing. The meristem was imaged in three anatomical planes. These include: the frontal plane, which bisects the dorsal and ventral sections of the plant body; the sagittal plane, which bisects the left and right sections; and the transverse plane, which bisects the apical and basal sections of the plant (Fig. 1A). The predicted apical cell was identified based on its geometry and its position in the central axis of the notch. Since it is unclear how far the proliferative zone of the meristem extends, we imaged multiple cell layers surrounding the predicted apical cell.

In the frontal plane, the stem cell niche of the mature meristem was positioned at the base of the notch (Fig. 1B-D). The apical cell was within the outer cell layer and appeared trapezoidal in this orientation (Fig. 1E,E′). The tissue morphology was identical on the left and right of the stem cell niche.

In the sagittal plane, the mature *M. polymorpha* thallus comprised many tissue layers. The roof of air chambers and gemma cups developed on the dorsal surface, while rhizoids formed on the ventral surface (Fig. 1F,G). The apical cell was triangular in the sagittal plane and surrounded by tissue protrusions (scales) on the ventral surface of the meristem (Fig. 1H-I′). The ventral side of the meristem was therefore morphologically distinct from the dorsal side of the meristem. The apical cell was located sub-apically in the outer cell layer, behind the apex of the thallus (green arrow, Fig. 1H-I′). The curved apical side of the meristem was therefore morphologically distinct from the thallus body on the basal side of the meristem.

In the transverse plane, the stem cell niche of the mature meristem was located on the ventral side of the thallus, above the scales (Fig. 1J-L). The stem cell niche was flanked by the left and right thallus lobes, and the apical cell was trapezoidal in this plane (Fig. 1M-M′). The tissue morphology was identical on the left and right of the meristem.

Imaging the meristem in three planes showed that the apical cell within the stem cell niche was trapezoid in the frontal and transverse planes, and triangular in the sagittal plane. Consistent with published results, the apical cell was therefore wedge shaped and likely divided in four directions based on its geometry (Fig. 1N). Our analysis indicated that the cellular anatomy of the mature meristem was identical on the left and right, but different on the dorsal and ventral sides, and different on the apical and basal sides of the meristem. We conclude that the 3D mature meristem has only one plane of symmetry – the sagittal plane – and is therefore bilaterally symmetrical.

### Reporter expression is distinct in each anatomical plane of the mature meristem, and is asymmetric in the dorsoventral and apical-basal axes

The maintenance of meristem structure in flowering plants involves the spatial restriction of gene expression to distinct domains within the meristem. Our analysis of the mature meristem showed that the apical cell is located on the ventral surface of the meristem, within the outer cell layer, and is neighboured by its daughter cells, which together form the stem cell niche (Fig. 1). To identify genes that are expressed in these discrete domains within the mature *M. polymorpha* meristem, we generated translational and transcriptional reporter lines for three candidate genes based on published literature.

**Fig. 2.**
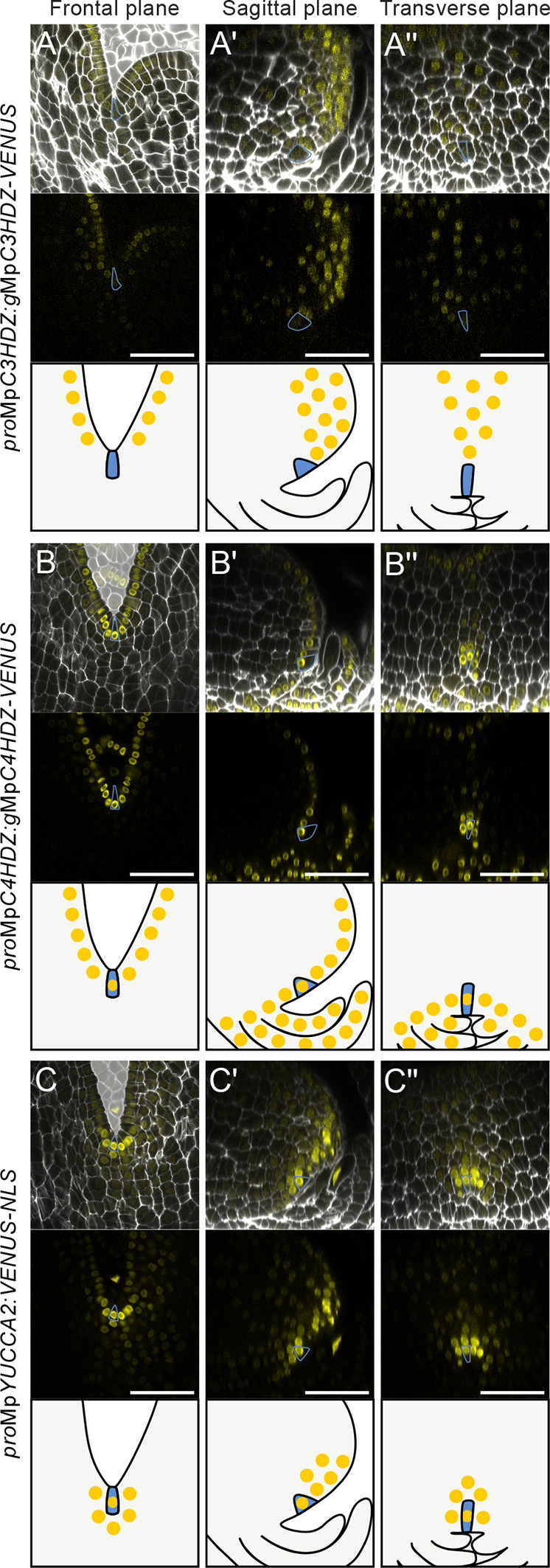
**Reporter expression is distinct in each anatomical plane of the mature meristem, and is asymmetric in the dorsoventral and apical-basal axes.** (A-C″) Meristems of reporter lines in the Tak-1×Tak-2 background grown for 4 weeks in white light. Lines include (A-A″) *pro*Mp*C3HDZ*:*g*Mp*C3HDZ*-*VENUS*, (B-B″) *pro*Mp*C4HDZ*:*g*Mp*C4HDZ*-*VENUS* and (C-C″) *pro*Mp*YUC2*:*VENUS*-*NLS*. SR 2200 cell wall stain is shown in white; VENUS signal is shown in yellow. In the frontal plane in the top panels, the space surrounding the meristem is marked in pale grey for clarity. In the middle panels, VENUS signal alone is shown. In the bottom panels, a schematic representation of the expression pattern is shown. The predicted apical cell is marked in blue throughout. *n*=10, *n*=10 and *n*=13 for A-A″, B-B″ and C-C″, respectively. All samples showed consistent expression patterns. (A,B,C) Frontal plane of the meristem. (A′,B′,C′) Optical reconstruction of the sagittal plane of the meristems in A, B and C. (A″,B″,C″) Optical reconstruction of the transverse plane of the meristems in A, B and C. A second independent line for each reporter is shown in Fig. S1. Scale bars: 50 µm.

To illustrate the asymmetry of stem cell niche position within the dorsoventral axis of the mature meristem, we imaged the mature meristems of Class III Homeodomain-Leucine-Zipper (C3HDZ) protein fusion lines reported in Wallner and Dolan, 2024 (Fig. 2A-A″, Fig. S1A-A″). C3HDZ proteins are required for meristem formation, meristem maintenance and adaxial-abaxial specification of leaf primordia in *Arabidopsis thaliana* (McConnell et al., 2001; Talbert et al., 1995), and MpC3HDZ localises to the dorsal meristem surface of *M. polymorpha* during meristem formation in the sporeling (Wallner and Dolan, 2024). In the sagittal plane of plants expressing *pro*Mp*C3HDZ*:*g*Mp*C3HDZ*-*VENUS* in the mature meristem, the MpC3HDZ-VENUS signal was restricted to the dorsal surface of the meristem, in the cell layers forming the air chambers (Fig. 2A′). Signal was stronger in daughter cells formed from a dorsal division of the apical cell, than in daughter cells derived from a ventral division of the apical cell. In the frontal plane, VENUS signal was weaker than the dorsal signal in the sagittal plane (Fig. 2A). Similarly, signal was low in the transverse plane and was only detectable above the apical cell (Fig. 2A″). We conclude that Mp*C3HDZ* reporter expression was enriched in the dorsal surface of the mature *M. polymorpha* meristem.

To visualise the outer cell layer, we generated Class IV HDZIP (C4HDZ) protein fusion lines (Fig. 2B-B″, Fig. S1B-B″), as C4HDZs are required for epidermal development in *A. thaliana*, and some family members, such as At*ATLM1* and At*PDF2*, are expressed in the L1 layer of the meristem (Abe et al., 2003; Nagata and Abe, 2023). In plants transformed with *pro*Mp*C4HDZ*:*g*Mp*C4HDZ*-*VENUS*, MpC4HDZ-VENUS signal was restricted to the outer cell layer in all three planes of the mature *M. polymorpha* meristem (Fig. 2B-B″). Within the outer cell layer, we observed stronger signal in the stem cell niche than in neighbouring cells (Fig. 2B-B″). MpC4HDZ-VENUS signal was also detected in scales that were one cell layer thick and developed from epidermal cells. MpC4HDZ-VENUS signal therefore marked the outer cell layer, including the stem cell niche.

To visualise the stem cell niche, we generated *YUCCA* (*YUC*) transcriptional reporter lines (Fig. 2C-C″, Fig. S1C-C″). *YUC* is a conserved auxin biosynthesis gene family in land plants and YUC proteins localise to sites of high auxin production, including the root apical meristem and shoot apical meristem of flowering plants, and the meristem of *M. polymorpha* (Blakeslee et al., 2019; Eklund et al., 2015; Hirakawa et al., 2020). In the frontal plane, *pro*Mp*YUC2*:*VENUS*-*NLS* reporter signal was high in the stem cell niche and neighbouring cells (Fig. 2C). The sagittal plane revealed that the strongest signal was detected in the dorsal region above the stem cell niche at the thallus apex (Fig. 2C′). We conclude that *pro*Mp*YUC2*:*VENUS*-*NLS* is highly expressed in a restricted region in and above the stem cell niche.

In summary, the VENUS signals of marker lines for MpC3HDZ, MpC4HDZ and Mp*YUC2* were spatially restricted to the dorsal surface, the outer cell layer (including the apical cell) and the stem cell niche of the mature meristem, respectively. Consistent with meristem anatomy (Fig. 1), reporter signal was asymmetric in the dorsoventral and apical-basal axes, but symmetric in the left-right axis. This expression asymmetry was only evident when examined in the frontal, sagittal and transverse planes.

### Dorsoventral anatomy and asymmetry is established from a symmetrical gemma meristem

**Fig. 3.**
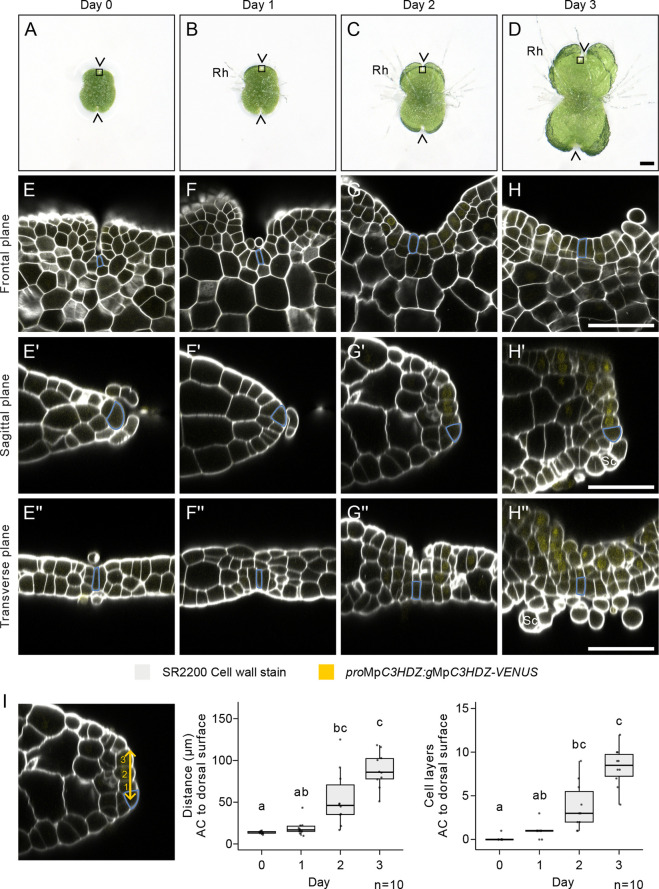
**Dorsoventral anatomy and asymmetry is established from a symmetrical gemma meristem.** (A-D) Keyence timelapse images of a Tak-1 *M. polymorpha* gemma for 3 days after transfer from gemma cup to media. (A) On day 0, immediately after transfer from the gemma cup, there are two notches on the gemma (arrowheads). (B) After 1 day, rhizoids (Rh) are formed from the dorsal and ventral surfaces. (C) On day 2, gemmaling size increases. (D) On day 3, notch depth increases, and new rhizoids are initiated from the ventral surface. Squares indicate the typical position of the meristems imaged and measured in E-I. (E-H) Confocal microscopy optical sections in the frontal plane of Tak-1 xTak-2 *pro*Mp*C3HDZ*:*g*Mp*C3HDZ*-*VENUS* gemmae on days 0-3. The predicted apical cell (blue) is located at the base of the notch in the outer cell layer at all time points. (E′-H′) A reconstruction of the sagittal plane of E-H. The predicted apical cell (blue) progressively moves from the apex (E′,F′) to the ventral side of the thallus (G′,H′). The thallus thickness increases, and ventral scales (Sc) form at day 3 (H′). (E″-H″) A reconstruction of the transverse plane of E-H. The predicted apical cell (blue) is located in the centre of the dorsoventral axis at day 0 (E″), but then localises to the ventral side of the thallus as the total thallus thickness increases (F″-H″). Scales (Sc) are evident in H″. *n*=10, *n*=6, *n*=12 and *n*=7 for E-E″, F-F″, G-G″ and H-H″, respectively. 0/10, 2/6, 10/12 and 7/10 samples showed reporter signal at day 0, day 1, day 2 and day 3, respectively. This experiment was repeated twice and showed consistent results. Furthermore, a second independent line is shown in Fig. S3. (I) Quantification of the distance (yellow line) and the number of cell layers (yellow numbers) from the predicted apical cell (AC, blue) to the dorsal meristem surface. G′ is re-used in I to explain how the distance and cell layer number were calculated. Kruskal–Wallis tests with Dunn's multiple comparisons were performed [χ^2^(3)=29.65, *P*<0.0001; χ^2^(3)=32.35, *P*<0.0001]. Box and whisker plots represent the median, the lower and upper quartiles, and the spread of the data. Lowercase letters indicate statistical difference (*P*<0.05). *n*=10 for each time point. The transgenic line used was the same as in Fig. S1A-A″. Scale bars: 200 µm in A-D; 50 µm in E-H″.

Mature *M. polymorpha* meristems are derived from meristems that develop on sporelings, or on small vegetative propagules known as gemmae. Gemmae form inside gemma cups on the dorsal surface of the thallus, where they remain dormant. After dispersal, the gemmae meristems are activated and gemmaling growth begins (Kato et al., 2020). It is unclear how the architecture of the meristem changes during gemmaling development and how this relates to the mature meristem. Therefore, we characterised the structure of the meristem during gemmaling development (Fig. 3).

Gemmae meristems were fixed immediately from the gemma cup (Day 0), and 1, 2 and 3 days after transfer to media (Fig. 3A-D). In the frontal plane, the stem cell niche was located at the base of the notch at all time points (Fig. 3E-H). The apical cell was rectangular and located in the outer cell layer, which formed a U-shaped invagination. The notch width expanded from day 2 as the meristem started to bifurcate (Fig. 3G-H, Fig. S2A-C). The tissue morphology on the left and right of the meristem was identical on each day.

In the sagittal plane, the ungerminated gemma (Day 0) was four cell layers thick with a subtending slime papilla above and below the apical cell (Fig. 3E′). The apical cell was positioned at the tip (apex) and the morphology of the tissue above the apical cell was identical to the tissue below the apical cell. Six out of 10 gemmae had one apparent apical cell, while four out of 10 gemmae had two apical cells within the same meristem, both equally sized and positioned at the apex (Fig. S2D,E). After 2 days, the stem cell niche was asymmetrically localised to one side of the apex (Fig. 3G′-H′).

To confirm that the stem cell niche was displaced to the ventral side of the apex, we examined the expression pattern of the *pro*Mp*C3HDZ*:*g*Mp*C3HDZ*-*VENUS* reporter, which marks the dorsal surface of the mature meristem (Fig. 2A-A″, Fig. S1). VENUS signal was undetectable at day 0, when the apical cell was positioned at the apex but was present in cells above the apical cell at day 1 to 2 (Fig. 3E′-H′, Figs S3, S4). The stem cell niche was therefore positioned on the ventral side of the gemmaling from day 1 to 2 (Fig. 3G′). Both the absolute distance and the number of cell layers between the apical cell and the dorsal meristem surface increased during days 1-3 (Fig. 3I).

In the transverse plane, the lobes to the left and right of the gemma meristem were two cell layers thick, and there were slime papillae above and below the apical cell (Fig. 3E″). The morphology of the tissue to the left and right of the apical cell was identical, and the morphology of the tissue above and below the apical cell was identical. During the next 2 days, more tissue layers were produced, and the stem cell niche was localised to the ventral side of the gemmaling (Fig. 3G″-H″). The ventral position of the stem cell niche was supported by dorsal expression of *pro*Mp*C3HDZ*:*g*Mp*C3HDZ*-*VENUS*. Furthermore, ventral structures, such as scales, were evident 3 days after removal from the gemma cup (Fig. 3H′).

These data indicate that the day 0 gemma meristem has left-right symmetry that persists through gemmaling development and into the mature meristem. However, unlike the mature meristem, the day 0 gemma meristem has dorsoventral symmetry. Anatomical dorsoventral asymmetry is detectable from day 1 to 2, and coincides with increased tissue layer formation and Mp*C3HDZ* reporter expression. We conclude that the dorsoventral anatomy and asymmetry that define the mature meristem are established during gemmaling growth.

### The stem cell niche is maintained in a sub-apical and ventral position after 1 week of growth

**Fig. 4.**
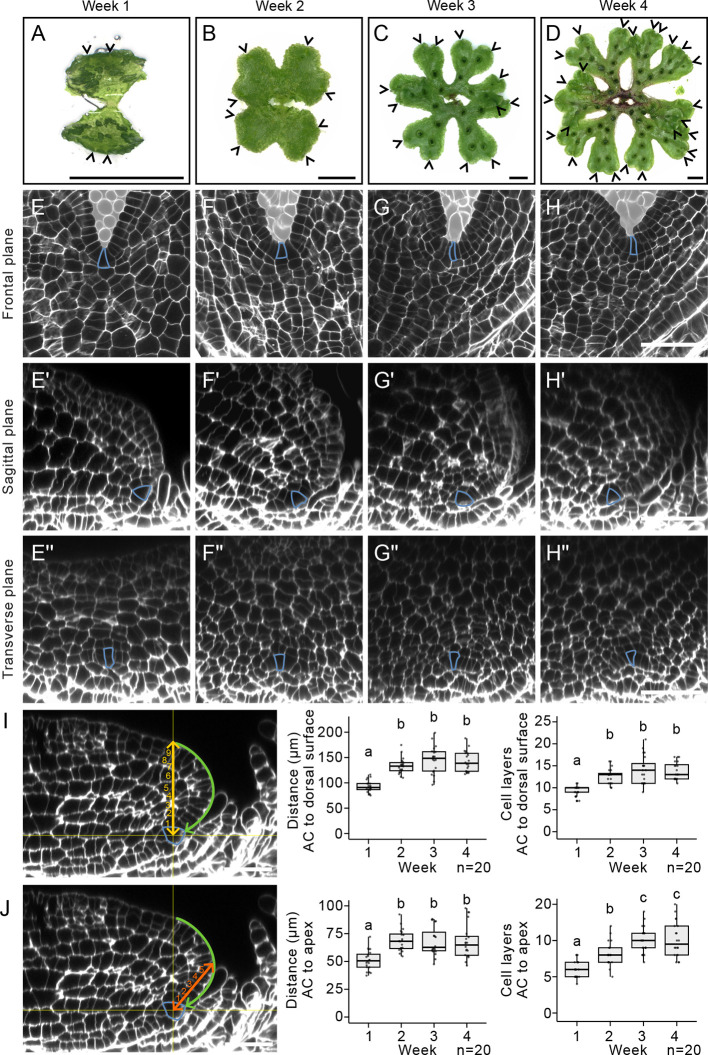
**The stem cell niche is maintained in a sub-apical and ventral position after 1 week of growth.** (A-D) Keyence images of Tak-1 *Marchantia polymorpha* plants grown for (A) 1, (B) 2, (C) 3 and (D) 4 weeks in white light after removal from the gemma cup. (E-H) Frontal plane of meristems. The predicted apical cells (blue) are located at the base of the notch, and meristem architecture is unchanged during this period. (E′-H′) Optical reconstructions of the sagittal plane of E-H. The predicted apical cells (blue) are located on the ventral side of the thallus body, behind the protruding thallus apex. (E″-H″) Optical reconstructions of the transverse plane of E-H. The predicted apical cells (blue) are located on the ventral side of the thallus and are surrounded by scales throughout. The space surrounding the meristem is marked in pale grey for clarity in E-H. The experiment was repeated twice with consistent results. (I) The vertical distance (yellow line) and the number of cell layers (yellow numbers) between the predicted apical cell (AC, blue) and the dorsal surface were measured. Welch's ANOVA tests with Games-Howell multiple comparisons were performed [F(3,40.61)=51.96, *P*<0.0001; F(3, 39.58)=40.7, *P*<0.0001]. Box and whisker plots represent the median, the lower and upper quartiles, and the spread of the data. *n*=20 for each time point. (J) The distance (orange line) and number of cell layers (orange numbers) between the predicted apical cell and the thallus apex (green arrow) were measured. Kruskal–Wallis tests with Dunn's multiple comparisons were performed [χ^2^(3)=25.74, *P*<0.0001; χ^2^(3)=41.03, *P*<0.0001]. Box and whisker plots represent the median, the lower and upper quartiles, and the spread of the data. *n*=20 for each time point. Lowercase letters indicate statistical difference (*P*<0.05). Scale bars: 5* *mm in A-D; 50 µm in E-J.

During the first 3 days of gemmaling development, the stem cell niche becomes displaced to the ventral surface and dorsoventral asymmetry is established (Fig. 3). However, the anatomy of the gemmaling meristem at day 3 differs from the mature meristem at week 4. To determine whether the overall anatomy of the meristem and the position of the stem cell niche stabilises in the mature plant, fixed meristem samples were cleared and imaged 1 week, 2 weeks, 3 weeks and 4 weeks after gemmae transfer to media (Fig. 4A-D).

In the frontal plane, the apical cell was located at the base of the notch throughout development (Fig. 4E-H). The apical cell was trapezoid and in the outer cell layer at each time point. We did not detect any differences in meristem anatomy in this plane between week 1 and 4. In the sagittal plane, the stem cell niche was ventrally and sub-apically localised, and was surrounded by scales throughout the 4-week time course (Fig. 4E′-H′). In the transverse plane, the stem cell niche was ventrally localised and surrounded by scales from weeks 1 to 4 (Fig. 4E″-H″). Therefore, the symmetry and arrangement of the meristem was consistent throughout the 4-week time course.

To determine if the relative ventral position of the stem cell niche changed during this time course, the vertical distance between the apical cell and the dorsal surface was measured at each time point (Fig. 4I). After an initial increase from weeks 1 to 2, there was no change in the total distance or number of cell layers between the apical cell and the dorsal surface from weeks 2 to 4 (Fig. 4I). To determine if the sub-apical position of the stem cell niche changed with age, the distance between the apical cell and the thallus apex was measured (Fig. 4J). The distance between the apical cell and the thallus apex increased from weeks 1 to 2 and then remained constant from week 2, whereas the number of cell layers remained constant from week 3 (Fig. 4J).

We conclude that the overall asymmetry and anatomy of the meristem was similar throughout weeks 1 to 4. The stem cell niche was positioned ventrally and sub-apically throughout weeks 1 to 4; however, its relative position within the meristem became stable 2-3 weeks after removal from the gemma cup. We conclude that the stem cell niche first becomes displaced to the ventral surface from day 2, before being displaced sub-apically between day 3 to 7.

## DISCUSSION

**Fig. 5.**
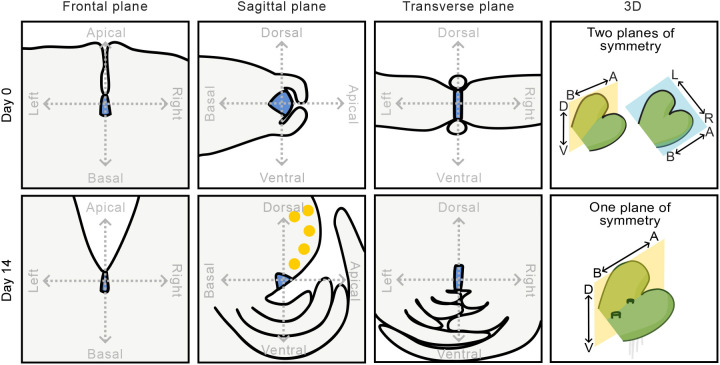
**Summary diagram of meristem anatomy in three anatomical planes at day 0 and day 14.** At day 0, the gemma meristem is notch shaped in the frontal plane, with left-right symmetry. In the sagittal plane, the meristem has dorsoventral symmetry. Both the left-right symmetry and the dorsoventral symmetry of the day 0 gemma are evident in the transverse plane. We conclude that there are two planes of symmetry at this timepoint, which correspond to the sagittal and frontal planes. During the next 14 days, the gemmaling meristem undergoes anatomical rearrangement. The left-right symmetry of the meristem is maintained; however, the dorsoventral symmetry is lost. Therefore, unlike the gemma meristem, the mature meristem has only one plane of symmetry in the sagittal plane. Both the anatomy and gene expression profiles of the mature meristem are asymmetrical: Mp*C3HDZ* reporter line signal (yellow) is restricted to the dorsal meristem surface only. We conclude that the gemmae meristem represents an intermediate stage of meristem development that, during the next 2 weeks, becomes the meristem of the mature *M. polymorpha* plant.

Meristems maintain a population of stem cells while producing cells that differentiate into tissues and organs. This balance of stemness and differentiation is achieved by spatially patterned gene expression networks that operate in the 3D anatomy of the meristem (Eshed Williams, 2021; Kohchi et al., 2021). We report the 3D structure of the mature *Marchantia polymorpha* meristem and show that the spatial localisation of MpC3HDZ, MpC4HDZ and Mp*YUC2* reporter signal is restricted to the dorsal surface, outer cell layer and stem cell niche of the meristem, respectively. The dorsoventral asymmetry of the mature meristem anatomy is consistent with the asymmetry of the reporter signal. We demonstrate how the 3D structure of the meristem changes during vegetative development. The cellular anatomy of the gemma meristem is relatively simple with dorsoventral symmetry. To generate the dorsoventral asymmetry that is characteristic of the mature meristem, the stem cell niche is displaced towards the ventral surface from day 2, and then becomes positioned sub-apically between days 3 and 7. The relative position of the stem cell niche within the meristem is stable after 2 weeks (Fig. 5).

The mature *M. polymorpha* meristem has left-right symmetry, dorsoventral asymmetry and apical-basal asymmetry. The meristem is therefore bilaterally symmetrical, unlike angiosperm shoot and root apical meristems, which are often radially symmetrical (Doerner, 2003; Moubayidin and Østergaard, 2015). The dorsoventral asymmetry of the *M. polymorpha* mature meristem is reflected in the asymmetrical expression of regulatory genes. For example, Mp*C3HDZ* reporter expression is low in the frontal plane but is strongly expressed in the dorsal domain of the meristem in the sagittal plane. This is consistent with published data showing that Mp*C3HDZ* regulates dorsoventral identity (Wallner and Dolan, 2024). Furthermore, enrichment of Mp*YUC2* reporter expression in the cells on the dorsal side of the stem cell niche is evident only in the sagittal plane. Previous reports of *pro*Mp*YUC2*:*GUS* expression showed that GUS signal was restricted to the notch, but did not show cellular level resolution or the restriction of expression to the dorsal surface (Eklund et al., 2015; Hirakawa et al., 2020; Takahashi et al., 2021). Our data indicate that the expression patterns of genes within the meristem are asymmetric, highlighting the necessity to examine all three planes to gain a complete picture of gene expression in the 3D meristem. Many GUS reporter lines for developmental genes in *Marchantia* have been shown to localise to the notch (Aki et al., 2019; Flores-Sandoval et al., 2015; 2018; Fu et al., 2024; Hirakawa et al., 2019; 2020; Liu et al., 2024), and our results provide a framework with which to identify their 3D expression in cellular detail in the meristem. These analyses will help to define the boundaries of the *M. polymorpha* meristem, by showing the spatial patterning of key meristem regulators.

Our temporal analysis of meristem anatomy from gemmae reveals that the meristem undergoes substantial change during the first 3 days of gemmaling development. This includes the establishment of dorsoventral asymmetry, which can develop in either orientation depending on which side the gemma lands after dispersal (Bowman, 2016; Mirbel, 1835). Light has been shown to polarise the gemma body; after the gemma lands, the surface towards the light source develops dorsal identity and the surface furthest away develops ventral identity (Bowman, 2016; Mirbel, 1835; Otto and Halbsguth, 1976). We found that Mp*C3HDZ* reporter signal was only detectable in the gemmaling 1-2 days after removal from the gemma cup. The timing of MpC3HDZ-VENUS reporter signal and asymmetry establishment correlates with early publications showing that dorsoventral polarity is irreversible after 1-3 days (Bowman, 2016; Mirbel, 1835; Pfeffer, 1871), and may correspond to light-induced polarity establishment. Changes in localisation of MpC3HDZ-VENUS during gemmaling development are also consistent with changing gene expression patterns during day 0 to day 3 for Mp*ERF20*, Mp*BZIP15* and Mp*C2H2-22* transcriptional reporter lines (Romani et al., 2024), and may be associated with the anatomical maturation of the meristem and the suppression of meristem dormancy. Our data are consistent with the hypothesis that the gemma meristem is an arrested meristem at an early stage of development. Upon removal from the gemma cup, gemma meristem development is re-initiated, leading to the formation of a mature meristem.

Our results provide the first spatio-temporal characterisation of meristem anatomy during the development of the haploid phase of *M. polymorpha*. We reveal that the meristem is itself dorsoventralised and this anatomical and molecular dorsoventrality is specified after dispersal from the gemmae cup, in response to the external environment. *M. polymorpha* grows horizontally, creating asymmetric external stimuli such as light along the dorsoventral body axis. This contrasts with other upright plants species such as *A. thaliana,* where the apical meristems are radially symmetrical, and the dorsoventrality of mature organs develops in primordia and not in the meristem. The diversity of meristem anatomy and symmetry in each lineage is therefore likely to reflect the myriad of terrestrial growth habits in land plants.

## MATERIALS AND METHODS

### Plant material and growth conditions

*Marchantia polymorpha* wild-type accessions Takaragaike-1 (Tak-1, male) and Takaragaike-2 (Tak-2 female), and transgenic plants were grown in continuous white light at 45 µmol m^−2^ s^−1^ at 23°C (see Table S1 for spectrum). Plants shown in Figs 1 and 2 were grown for 2 weeks on plates containing ½ B5 Gamborg's Media [½ strength B5 Gamborg+0.5 g/L MES hydrate+1% sucrose+1% plant agar (pH 5.5)] and then transferred to SacO2 Microboxes containing autoclaved 3:1 compost:vermiculite mix for a further 2 weeks. Plants in Fig. 3 were grown for 3 days on media plates, and plants in Fig. 4 were grown for 4 weeks on media plates without transfer to soil. To generate spores for transformation, 2-week-old plants grown on media plates in continuous white light were transferred to soil and moved to 16 h far-red enriched light (50 µmol m^−2^ s^−1^, see Table S1 for spectrum), 8 h dark, at 20°C. Plants were crossed and the mature sporangia harvested. Sporangia were dried for 4 weeks before freezing at −70°C.

### Generation of plasmids for transformation

To generate translational reporter constructs, we followed the GreenGate protocol (Lampropoulos et al., 2013). *pro*Mp*C3HDZ*:*g*M*pC3HDZ*-*VENUS* lines were generated by Wallner and Dolan (2024). A 4309 bp region upstream of the ATG start codon was defined as the Mp*C4HDZ* promoter. The genomic gene sequence of Mp*C4HDZ* encompassed 3627 bp (including exons and introns without the stop codon). The MpC4HDZ promoter was amplified in one piece and the Mp*C4HDZ* genomic gene sequence was amplified in three pieces from Tak-1 DNA by CloneAmp HiFi PCR Premix with primers as listed in Table S1, and cloned via BsaI restriction sites into GreenGate entry modules with a pUC19-based vector backbone. To generate translational fusion *pro*Mp*C4HDZ*:*g*M*pC4HDZ*-*VENUS*, GreenGate entry modules listed in Table S1 were assembled into the pGreen-IIS based destination vector. The Mp*YUC*2 promoter was defined as the 4001 bp region upstream of the ATG start codon and amplified from Tak-1 DNA with primers listed in Table S1. Since it contained internal BsaI restriction sites identical to the GreenGate overhangs, the transcriptional reporter construct *proMpYUC2:VENUS-linker-NLS* was generated by Gibson Assembly using a custom-made GreenGate destination vector backbone. The linker-VENUS module has been published previously (Wallner et al., 2023) and the chlorsulfuron plant resistance module was adapted from the OpenPlant toolkit (Sauret-Güeto et al., 2020). Cloning was performed by the Protein Technologies Facility at the Vienna BioCenter.

### Generation of transgenic lines

Spores generated from a Tak-1×Tak-2 cross were transformed with reporter constructs according to the protocol described by Wallner and Dolan (2024) (adapted from Ishizaki et al., 2008). Sporelings were plated on ½ B5 Gamborg's Media containing 0.5 µM chlorsulfuron and 100 mg/L cefotaxime. Positive transformants were grown on non-selective plates and screened for reporter expression. At least 10 independent lines were screened, and four lines (two males and two females) were selected that showed consistent and strong reporter signal. Reporter expression in one independent line is shown in Fig. 2, and a second independent line is shown in Fig. S1. The lines were verified using the ‘Plant sequencing primers’ listed in Table S1. Plants were stored as gemmae stocks in media stabs at 4°C, and stored on media plates as mature plants in 8 h white light (45 µmol m^−2^ s^−1^, see Table S1 for spectrum), 16 h dark, at 17°C.

### Tissue fixation and clearing

The notches of plants were harvested and transferred immediately to 10% formalin solution (10% neutral buffered, 4% w/v formaldehyde)+0.1% Brij L23 solution (protocol adapted from Mulvey and Dolan, 2023). Samples were vacuumed twice for 25 min each, and then washed three times in phosphate-buffered saline (PBS). PBS solution was replaced with Clear-See α solution [10% (w/v) xylitol+15% (w/v) sodium deoxycholate+25% (w/v) urea+50 mM sodium sulphite anhydrous]. Samples were vacuumed for 25 min, and then incubated in the dark on a shaker overnight in Clear-See α solution. The next day, the solution was replaced, and samples were incubated in the dark on a shaker until imaging (minimum 5 days).

### Imaging and microscopy

Live plants were imaged using the Keyence VHX-7000 digital microscope with the VH-ZST and VH-Z00R/W/T objectives. For fixed samples, 0.2% SR 2200 cell wall stain was added to cleared samples the day before imaging. Samples were mounted on glass slides in Clear-See α solution in 0.25* *mm thick Gene Frames, and imaged using an inverted point scanning Zeiss LSM880 confocal microscope with a 40×/1.2 LD LCI plan-apochromat, water, glycerol DIC AutoCorr objective and MBS 458/514 and MBS −405 filters. Silicon oil was used for immersion and *z*-stacks of 300-600 slices were acquired depending on the thickness of the sample. The optimal *z*-stack interval was selected. To detect SR 2200 signal, a 405 nm diode laser (1-2%) was used for excitation, and signal was detected between 419 and 499 nm with a 32 µm pinhole (∼1 AU). To detect VENUS signal, a 514 nm argon laser (20% for MpC3HDZ and MpC4HDZ, and 8% for Mp*YUCCA2*) was used for excitation and VENUS signal was detected between 526 and 579 nm with a 39 µm pinhole (∼1 AU). The two channels were scanned sequentially in all imaging. For images shown in Fig. 2 and Fig. S1, the track was switched after each frame, whereas for Fig. 3 and Fig. S3 the track was switched after each *z*-stack.

For Fig. 1H,L, week 4 samples stained with SR 2200 were imaged using a Zeiss Z1 Light-sheet microscope. Samples were mounted in 3% Low Melting Agarose in Clear See solution and incubated at 4°C for 1-3 h. An agarose block containing the sample was cut and glued to a plastic support, which was attached to a capillary tube held within the sample mount. A large sample imaging chamber (*n*=1.33-1.58) was filled with Clear See solution. LSFM 5×/0.1 foc excitation objectives were used with a EC-Plan Neofluar 5×/0.16 foc detection objective. A 405 nm 20 mW laser (5%) was used to detect SR 2200 signal.

### Image analysis

The *z*-stacks acquired were used to reconstruct the sagittal and transverse planes using Orthogonal Views in Fiji. Stacks were rotated to produce sagittal planes through the geometric centre of the notch. For Figs 3 and 4, the sagittal plane was measured at all stages of the bifurcation cycle. The distance between the apical cell and the thallus apex, and the distance between the apical cell and the dorsal surface were measured. Cell layers were counted along these measurement axes; a cell layer was counted each time a new cell wall was crossed. Meristems that had recently produced gemma cups were excluded from the analysis. All meristems shown in figures had recently bifurcated and the central lobe had expanded. This stage was chosen because the next round of bifurcation (and thus stem cell duplication) was least likely to be occurring. Experiments were performed once, unless stated otherwise in the figure legend.

### Statistical analysis and data presentation

All statistical analysis was performed using R-studio. Welch's ANOVA tests with Games–Howell multiple comparisons were used when data were normally distributed with unequal variance. When data were not normally distributed, Kruskal–Wallis with Dunn's multiple comparisons tests were used. All figures were made using Inkscape.

### Reagents, equipment and software

Further manufacturer and identification details for all consumables and equipment can be found in Table S1.

## Supplementary Material



10.1242/develop.204349_sup1 Supplementary information

Table S1. Table including light spectra and lists of reagents, resources, plasmids and primers
