# Supplementary material for: Three-dimensional anatomy and dorsoventral asymmetry of the mature Marchantia polymorpha meristem develops from a symmetrical gemma meristem
Source: Development. 2024 Nov 29;151(23):dev204349. doi: 10.1242/dev.204349 (PMC11634034; doi:10.1242/dev.204349)
Supplement: Supplementary information [file develop-151-204349-s1.pdf]

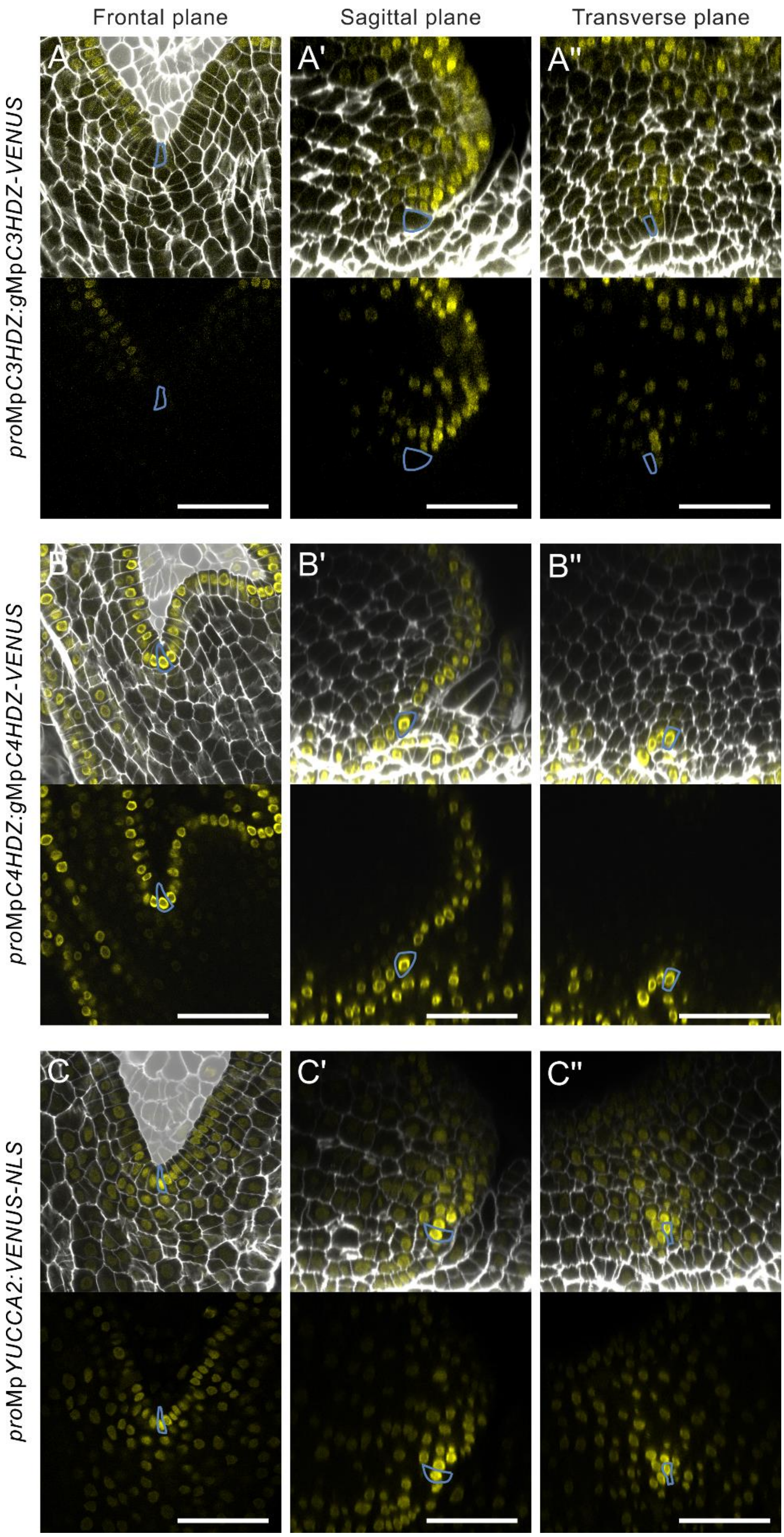

Development • Supplementary information

Fig. S1. MpC3HDZ, MpC4HDZ and Mp*YUC2* reporter line expression

(**A**-**C''**) Meristems of a second independent reporter line in the Tak-1 x Tak-2 background grown for four weeks in white light. Lines show the same expression patterns as Fig. 2 for (**A**-**A''**) *pro*Mp*C3HDZ*:*g*Mp*C3HDZ-VENUS*, (**B**-**B''**) *pro*Mp*C4HDZ*:*g*Mp*C4HDZ-VENUS*, and (**C**-**C''**) *pro*Mp*YUC2*:*VENUS-NLS*. SR 2200 cell wall stain is shown in white, and VENUS signal is shown in yellow. In the top panels, the space surrounding the meristem is marked in pale grey for clarity. In the bottom panels, VENUS signal alone is shown. The predicted apical cell is marked in blue throughout. n=3, n=9, n=6 for A-A'', B-B'' and C-C'' respectively. All samples showed consistent expression patterns. (**A, B, C**) Frontal plane of the meristem. (**A'**, **B'**, **C'**) Optical reconstruction of the sagittal plane of the meristems in A, B, C. (**A''**, **B''**, **C''**) Optical reconstruction of the transverse plane of the meristems in A, B, C. Scale bars: A-C''= 50 µm.

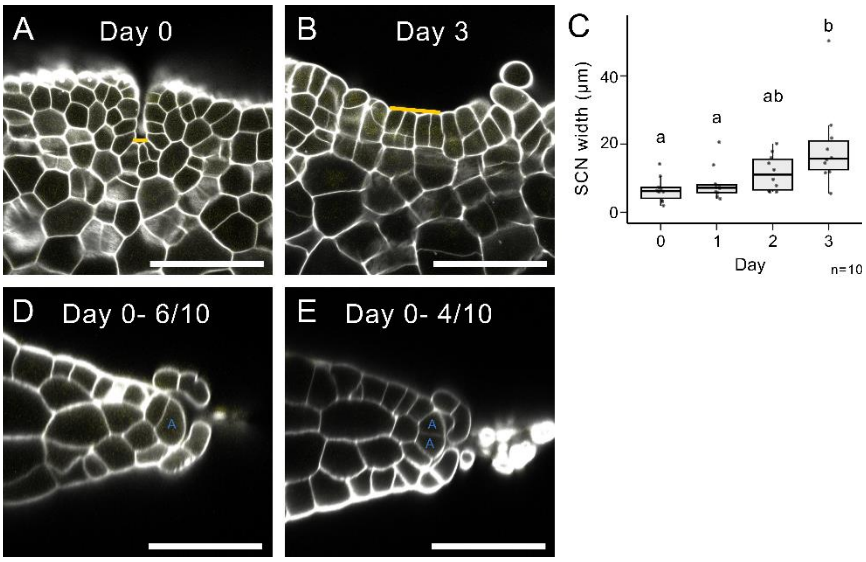

Fig. S2. Stem cell niche width increases during day 0-3 of gemmaling development

(**A**-**C**) Measurements of the stem cell niche width, as shown with a yellow line. Cells were included if they were located in the notch base, were smaller than the surrounding cells, and trapezoid/ rectangular in shape. Panels A and B are the images in Fig. 3E and 3H respectively. A Kruskal-Wallis test with Dunn's multiple comparison was performed [$\chi^2(3)=13.15$, $p=0.0043$]. Lower case letters denote statistical difference with a p-value of <0.05. n= 10 for each time point. (**D**-**E**) Day 0 gemma meristems had either one (D) or two (E) detectable apical cells (blue A). 4/10 samples had two apical cells, that were equal in size and both located at the apex. Panel D is the image in Fig. 3E'. Scale bars: A-B, D-E= 50 µm.

Development • Supplementary information

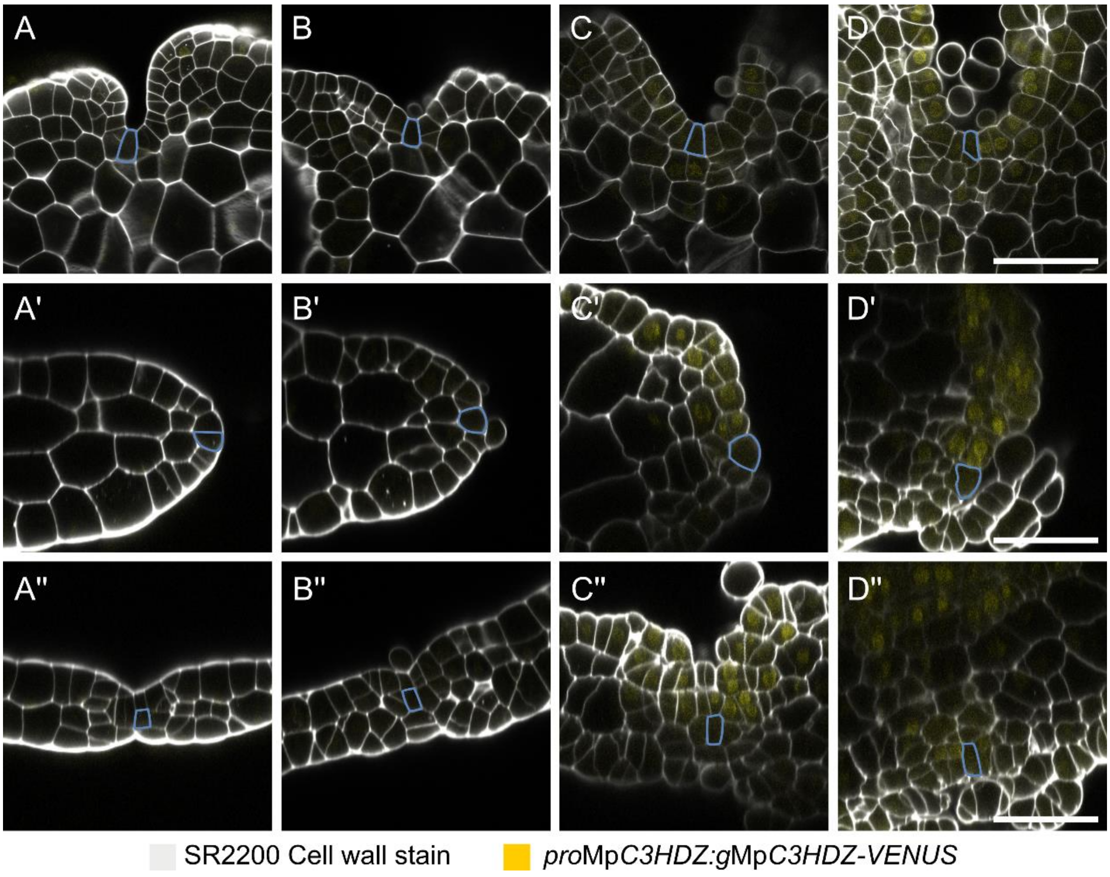

Fig. S3. MpC3HDZ reporter line signal in gemmae at day 0 to day 3

(**A**-**D**) Optical sections in the frontal plane of gemmae on day 0-3. This second independent Tak-1 xTak-2 *pro*Mp*C3HDZ*:*g*Mp*C3HDZ-VENUS* line shows consistent reporter signal as the line presented in Fig. 3. The predicted apical cell is shown in blue. (**A'**-**D'**) A reconstruction of the sagittal plane of A-D. (**A''**-**D''**) A reconstruction of the transverse plane of A-D. n= 10, n= 10, n=10 and n=9 for A-A'', B-B'', C-C'' and D-D'' respectively. 2/10, 7/10, 10/10 and 9/9 samples showed reporter signal at each time point. Plant morphology is shown in Fig. S4. Scale bars = 50 µm.

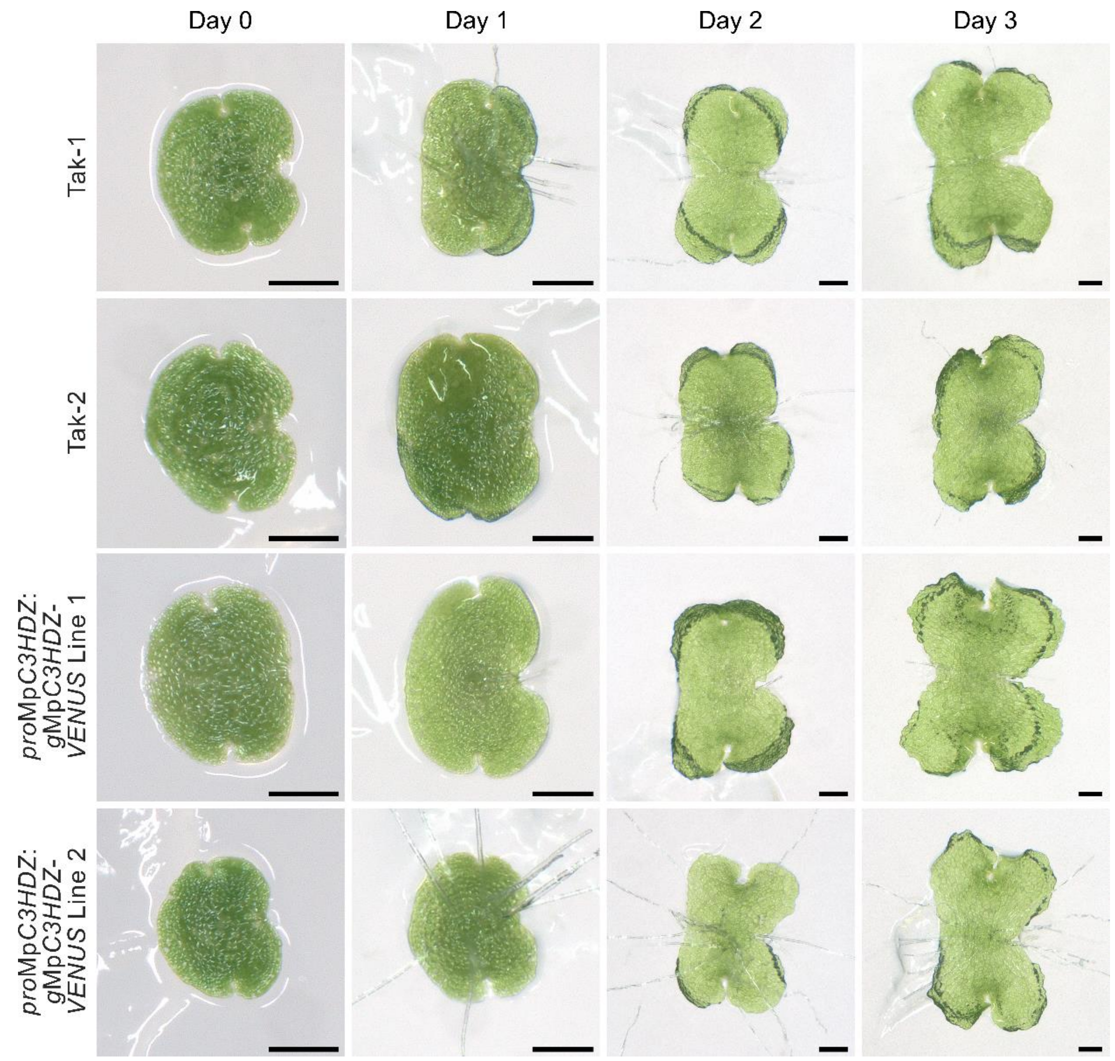

Fig. S4. Morphology of wild type gemmae and MpC3HDZ reporter lines from day 0 to day 3

Keyence images of typical gemmae for MpC3HDZ translational reporter lines. Plants were imaged 0, 1, 2 and 3 days after removal from the gemmae cup. Both wild type (Tak-1 and Tak-2) and the two independent *pro*Mp*C3HDZ*:*g*Mp*C3HDZ-VENUS* lines are morphologically similar. Line 1 is shown in Fig. 3 and Fig. S1 and Line 2 is shown in Fig. 2 and Fig. S3. Scale bars= 250µm.

Table S1. Table including light spectra and lists of reagents, resources, plasmids and primers

Available for download at
https://journals.biologists.com/dev/article-lookup/doi/10.1242/dev.204349#supplementary-data
