# Supplementary material for: Three-dimensional anatomy and dorsoventral asymmetry of the mature Marchantia polymorpha meristem develops from a symmetrical gemma meristem
Source: Development. 2024 Nov 29;151(23):dev204349. doi: 10.1242/dev.204349 (PMC11634034; doi:10.1242/dev.204349)
Supplement: Supplementary file 1 [file develop-151-204349_review_history.pdf]

Three-dimensional anatomy and dorsoventral asymmetry of the mature *Marchantia polymorpha* meristem develops from a symmetrical gemma meristem

Victoria Spencer, Eva-Sophie Wallner, Katharina Jandrasits, Natalie Edelbacher, Magdalena Mosiolek and Liam Dolan

DOI: 10.1242/dev.204349
Editor: Dominique C Bergmann

Review timeline
Original submission: 23 August 2024
Editorial decision: 4 October 2024
First revision received: 29 October 2024
Accepted: 3 November 2024

Original submission

First decision letter

MS ID#: DEVELOP/2024/204349

MS TITLE: 3D anatomy and dorsoventral asymmetry of the mature *Marchantia polymorpha* meristem develops from a symmetrical gemma meristem

AUTHORS: Liam Dolan, Victoria Spencer, Eva-Sophie Wallner, Katharina Jandrasits, Natalie Edelbacher and Magdalena Mosiolek

I have now received all the referees reports on the above manuscript, and have reached a decision. The referees' comments are appended below, or you can access them online: please go to.

As you will see, the reviewer consider this foundational work for the field. They differ in the extent to which they consider its primarily descriptive nature a strength or weakness, but on balance the clarity of text and figure presentation makes this a valuable addition to the literature. Each reviewer does have some reasonable suggestions for edits that will improve the clarity of the message and these should be covered in a revision.

The overall evaluation is positive and we would like to publish a revised manuscript in Development, provided that the referees' comments can be satisfactorily addressed. Please attend to all of the reviewers' comments in your revised manuscript and detail them in your point-by-point response. If you do not agree with any of their criticisms or suggestions explain clearly why this is so. If it would be helpful, you are welcome to contact us to discuss your revision in greater detail. Please send us a point-by-point response indicating your plans for addressing the referees' comments, and we will look over this and provide further guidance.

Reviewer 1

Advance summary and potential significance to field

© 2024. Published by The Company of Biologists under the terms of the Creative Commons Attribution License (https://creativecommons.org/licenses/by/4.0/).

Meristems play a crucial role in plant development. Although the anatomy and molecular regulation of meristems in flowering plants are well understood, the corresponding mechanisms in non-flowering plants, particularly bryophytes, remain largely unexplored. In this study, the authors characterize the three-dimensional architecture of meristems at cellular resolution in the model thalloid liverwort Marchantia polymorpha during thallus development originating from mature gemmae. Additionally, the authors demonstrate the spatiotemporal expression patterns of several reporter genes, including MpC3HDZ, MpC4HDZ, and MpYUC2, and clearly illustrate the establishment of dorsoventral asymmetry in early gemmaling development within the first two days. Recent advances have expanded our understanding of the molecular regulators governing haploid meristem function in Marchantia. Consequently, this study provides a foundational framework for elucidating the development of the gametophyte body in Marchantia.
The manuscript is generally clear and well-written, with figures that are presented effectively. To enhance the manuscript further, I suggest that the authors revise it in response to the following points.

Comments for the author

General points
Several studies have investigated the cellular organization and establishment of leafy gametophyte meristems in the moss Physcomitrium patens. In addition to thalloid species, many liverworts possess leafy gametophytes with symmetric or asymmetric dorsoventral axes. It would be valuable to concisely introduce and/or discuss the commonalities and differences in meristem structure and development among haploid gametophytes across various bryophytes.

Minor points
Line 91-93, "The out cell layer on the apical sideâ€¦on the basal side of the stem cell niche." It is not clear where the parallel dorsal structures are specified in the Figure 1.

Line 141, Fig. S1C-C", The signal of the proMpYUC2 reporter in Figure S1C-C" appears slightly broader compared to that in Figure 2C-C". Please describe the differences in the signal patterns between Figure S1C-C" and Figure 2C-C", and provide a rationale for the interpretation presented in Figure 2C-C".

Legend of Figure 3, panelI, "Fig. 2G' is shown in panelI to explain how the distance and cell layer number were calculated." Fig. 2G' must be Fig. 3G'

<u>Reviewer 2</u>

Advance summary and potential significance to field

This study examined the three-dimensional structure of the meristem in the liverwort Marchantia polymorpha at various developmental stages. The most important finding is the asymmetry of the meristem along the dorsal-ventral plane. The refined anatomy of the meristem can be used as a good reference for studies concerning stem cell maintenance and development in this plant species.

Comments for the author

The experiments were all well done and the results were clearly presented. I am just not sure about the significance of the asymmetry of the meristem. What is the significance of this asymmetry in terms of plant growth and adaptation to a terrestrial habitat?

Also, there and no mechanism and explanation how this asymmetry was formed.

In the beginning, the authors raised the question of "our understanding of how meristem architecture is regulated in non-flowering plants such as bryophytes is lacking", but they provided no answer to this question. How the stem cell niche is displaced towards the ventral surface from day 2? By more frequent cell division of the cells in the apical cell, stem cells or meristem cells?

© 2024. Published by The Company of Biologists under the terms of the Creative Commons Attribution License (https://creativecommons.org/licenses/by/4.0/).

Minor issues:

Is the result in Fig 2A published in Wallner & Dolan, 2024. If so, cite the report but do not repeat here.

Fig 4. How is the meristem is defined? Is the apical cell the center of the meristem, if not, which looks like the case, why use the apical cell as the proxy of meristem?

Reviewer 3

Advance summary and potential significance to field

In this paper, Spencer et al perform lovely imaging, including of reporter lines, to determine the 3d structure of the Marchantia polymorpha meristem. This paper included gorgeous optical sections of Marchantia, and careful analysis of gemmaling development. This foundational work has the potential to be very useful in the field of plant development, especially as Marchantia gains steam as a developmental model system. However, the manuscript needs some revisions to be more approachable to the general reader.

Comments for the author

My suggestions are fairly minor. Specifically:
It was never clear to me how the decision was made as to what was the meristem and what was not the meristem, other than the apical cell. More labeling of the figures, especially of figure 1, would be very helpful for the reader to understand what the limits of the meristem are. Information on how this decision was made would also be helpful.
The shift from symmetry to asymmetry shown in Fig. 3 was interesting, but I was left wondering where the extra cell layers (i.e. those shown in H' and H") came from. Are these generated by the meristem? Or are other cell layers proliferating and driving the asymmetry? I think the authors think the latter is happening, based on their statement that (line 273) 'dorsoventrality is specified by the environment', with 'the environment' being the tissues around the meristem. The manuscript would benefit from a discussion of this point, including what they mean by 'the environment' if my interpretation is incorrect.

- Additional minor comments.
Fig 1 edits: (a) Labeling the apical end of the apical cell in Figure 1N would make interpretation considerably easier; (b) the illustrations in Fig. 1A would be easier to interpret if the notch was oriented the same way it is in Fig. 1B-E'

First revision

Author response to reviewers' comments

Reviewer 1: SUMMARY OF THE ADVANCE MADE IN THIS PAPER AND ITS POTENTIAL SIGNIFICANCE TO THE FIELD
Meristems play a crucial role in plant development. Although the anatomy and molecular regulation of meristems in flowering plants are well understood, the corresponding mechanisms in non-flowering plants, particularly bryophytes, remain largely unexplored. In this study, the authors characterize the three-dimensional architecture of meristems at cellular resolution in the model thalloid liverwort Marchantia polymorpha during thallus development originating from mature gemmae. Additionally, the authors demonstrate the spatiotemporal expression patterns of several reporter genes, including MpC3HDZ, MpC4HDZ, and MpYUC2, and clearly illustrate the establishment of dorsoventral asymmetry in early gemmaling development within the first two days. Recent advances have expanded our understanding of the molecular regulators governing haploid meristem function in Marchantia.

© 2024. Published by The Company of Biologists under the terms of the Creative Commons Attribution License (https://creativecommons.org/licenses/by/4.0/).

Consequently, this study provides a foundational framework for elucidating the development of the gametophyte body in Marchantia.
The manuscript is generally clear and well-written, with figures that are presented effectively. To enhance the manuscript further, I suggest that the authors revise it in response to the following points.

SUGGESTIONS TO AUTHORS
Several studies have investigated the cellular organization and establishment of leafy gametophyte meristems in the moss Physcomitrium patens. In addition to thalloid species, many liverworts possess leafy gametophytes with symmetric or asymmetric dorsoventral axes. It would be valuable to concisely introduce and/or discuss the commonalities and differences in meristem structure and development among haploid gametophytes across various bryophytes.

Response: We have included more information about moss meristems and leafy liverworts in the introduction (Line 47-50). We hope this highlights the diversity of stem cell arrangement and cell division within the gametophyte stage of bryophytes.

Line 91-93, "The outer cell layer on the apical side…on the basal side of the stem cell niche." It is not clear where the parallel dorsal structures are specified in the Figure 1.

Response: This paragraph has been re-written for clarity (Line 98-105).

Line 141, Fig. S1C-C", The signal of the proMpYUC2 reporter in Figure S1C-C" appears slightly broader compared to that in Figure 2C-C". Please describe the differences in the signal patterns between Figure S1C-C" and Figure 2C-C", and provide a rationale for the interpretation presented in Figure 2C-C".

Response: We have repeated the fixation and analysis of the two independent *pMpYUCCA2::VENUS-NLS* lines at week 4 (Figure 2 and Sup Figure 1). The results were consistent with those shown previously; VENUS signal is enriched around the stem cell niche and in the adjacent dorsal surface of the meristem. We replaced the images in Figure 2 and Sup Figure 1 with images that show this localisation more clearly. Slight differences between samples are likely to reflect differences in the developmental stage of the meristem, but the overall localisation pattern is consistent. We have also replaced the blue shading of the apical cell with a blue outline in all figures that show reporter signal. This is because the shading was masking the reporter line signal in the apical cell.

Legend of Figure 3, panel I, "Fig. 2G' is shown in panel I to explain how the distance and cell layer number were calculated." Fig. 2G' must be Fig. 3G'
Response: This has been corrected.

Reviewer 2: SUMMARY OF THE ADVANCE MADE IN THIS PAPER AND ITS POTENTIAL SIGNIFICANCE TO THE FIELD

This study examined the three-dimensional structure of the meristem in the liverwort Marchantia polymorpha at various developmental stages. The most important finding is the asymmetry of the meristem along the dorsal-ventral plane. The refined anatomy of the meristem can be used as a good reference for studies concerning stem cell maintenance and development in this plant species.

SUGGESTIONS TO AUTHORS

The experiments were all well done and the results were clearly presented. I am just not sure about the significance of the asymmetry of the meristem. What is the significance of this asymmetry in terms of plant growth and adaptation to a terrestrial habitat?

Response: Unlike many well studied model species such as *Arabidopsis thaliana*, *Marchantia polymorpha* develops a prostrate growth habit and grows horizontally along the ground. This dorsoventral asymmetry in the external environmental likely drives the anatomical and

© 2024. Published by The Company of Biologists under the terms of the Creative Commons Attribution License (https://creativecommons.org/licenses/by/4.0/).

molecular asymmetry of the meristem described here, to produce specialised tissue types in response to differential external stimuli such as light. We have added this discussion point to the final paragraph of the text (line 283-290).

Also, there and no mechanism and explanation how this asymmetry was formed.

Response: Our intention with this paper was to provide a spatio-temporal framework on which future studies can elucidate the molecular mechanisms by which meristem asymmetry is formed and maintained.

In the beginning, the authors raised the question of "our understanding of how meristem architecture is regulated in non-flowering plants such as bryophytes is lacking", but they provided no answer to this question.

Response: We hope our work is foundational for future studies to identify how meristem architecture is regulated. However, we agree that we do not answer this question, and have reworded the introduction to prevent confusion about the intention of our paper (line 42).

How the stem cell niche is displaced towards the ventral surface from day 2? By more frequent cell division of the cells in the apical cell, stem cells or meristem cells?

Response: This is a very good question. In future research, we would like to generate a live-imaging approach in which we can track cell divisions and potentially answer this question. However for now, this is beyond the scope and experimental limits of our study.

Minor issues:

Is the result in Fig 2A published in Wallner & Dolan, 2024. If so, cite the report but do not repeat here.

Response: The mature meristem was not presented in Wallner and Dolan, 2024. The oldest sample shown in the paper was a day 9 sporeling.

Fig 4. How is the meristem is defined? Is the apical cell the center of the meristem, if not, which looks like the case, why use the apical cell as the proxy of meristem?

Response: We don't know how large the meristem is and it likely changes during development. We use the apical cell because it is a constant landmark, and the ultimate source of all cells in the meristem. We have added a sentence to clarify that the bounds of the meristem are currently unknown (line 92-94) and a sentence to suggest how the meristem limits may be defined in the future (line 263-264).

Reviewer 3: SUMMARY OF THE ADVANCE MADE IN THIS PAPER AND ITS POTENTIAL SIGNIFICANCE TO THE FIELD

In this paper, Spencer et al perform lovely imaging, including of reporter lines, to determine the 3d structure of the Marchantia polymorpha meristem. This paper included gorgeous optical sections of Marchantia, and careful analysis of gemmaling development. This foundational work has the potential to be very useful in the field of plant development, especially as Marchantia gains steam as a developmental model system. However, the manuscript needs some revisions to be more approachable to the general reader.

SUGGESTIONS TO AUTHORS

My suggestions are fairly minor. Specifically:

It was never clear to me how the decision was made as to what was the meristem and what was not the meristem, other than the apical cell. More labeling of the figures, especially of figure 1, would be very helpful for the reader to understand what the limits of the meristem are. Information on how this decision was made would also be helpful.

Response: We include a definition of the meristem in line 82-83, stating that the meristem

© 2024. Published by The Company of Biologists under the terms of the Creative Commons Attribution License (https://creativecommons.org/licenses/by/4.0/).

includes both the stem cell niche and the surrounding proliferative zone.
However, we agree with the reviewer that this is not clearly defined in Marchantia. We hope that our work will provide a foundation for future studies to specify these zones within the mature meristem in each plane. Our imaging includes the apical cell (which is definitely in the meristem), and the surrounding tissue, including differentiating structures such as scales and early forming air chambers, which likely mark the boundary of the meristem. We added a sentence to clarify that the bounds of the meristem are currently unknown (line 92-94) and a sentence to suggest how the meristem limits may be defined in the future (line 263-264).

The shift from symmetry to asymmetry shown in Fig. 3 was interesting, but I was left wondering where the extra cell layers (i.e. those shown in H' and H") came from. Are these generated by the meristem? Or are other cell layers proliferating and driving the asymmetry?
Response: We agree that the creation of new cell layers during asymmetry establishment is an interesting point. In future research, we would like to generate a live-imaging approach in which we can track cell divisions and potentially answer this question. However, this is beyond the scope and experimental limits of our study.
I think the authors think the latter is happening, based on their statement that (line 273) 'dorsoventrality is specified by the environment', with 'the environment' being the tissues around the meristem. The manuscript would benefit from a discussion of this point, including what they mean by 'the environment' if my interpretation is incorrect.
Response: We thank the reviewer for highlighting the lack of clarity in our statement. Line 273 “specified by the environment” was referring to the external environment in which the gemma is located after dispersal from the gemma cup. We have updated the text to clarify this point (Line 283-285).

- Additional minor comments.
Fig 1 edits: (a) Labeling the apical end of the apical cell in Figure 1N would make interpretation considerably easier;
Response: We have added labels to the diagram as suggested.

(b) the illustrations in Fig. 1A would be easier to interpret if the notch was oriented the same way it is in Fig. 1B-E'
Response: We have remade the illustrations in Figure 1A as suggested.

In addition to the reviewers’ comments, we have made further minor changes to improve the manuscript.

- Images in Figure 3 are of Tak-1, not the C3HDZ reporter line as stated. This has been corrected and image of C3HDZ added to the supplementary data (Sup. Figure 4).
- We repeated the C3HDZ gemmae analysis experiment in Figure 3 E-H’’, which showed consistent results with those reported in this figure (line added to Figure legend).
- We added a second independent C3HDZ reporter line imaged at day 0-3 to the supplementary data. This again confirms the results shown in Figure 3. In both independent lines, some samples at day 1 show no expression whilst others show weak reporter signal in the dorsal surface. We have therefore adjusted the text to state that signal is detectable from “day 2” to “day 1-2” to be more accurate.
- We added the primer information for the generation of constructs which we mistakenly omitted from the submitted version (Supplementary table and Lines 305-318).
- We swapped the reference for Wallner and Dolan, 2024 from the preprint to the recently published manuscript.

Second decision letter

© 2024. Published by The Company of Biologists under the terms of the Creative Commons Attribution License (https://creativecommons.org/licenses/by/4.0/).

MS ID#: DEVELOP/2024/204349

MS TITLE: 3D anatomy and dorsoventral asymmetry of the mature *Marchantia polymorpha* meristem develops from a symmetrical gemma meristem

AUTHORS: Liam Dolan, Victoria Spencer, Eva-Sophie Wallner, Katharina Jandrasits, Natalie Edelbacher and Magdalena Mosiolek

I am happy to tell you that your manuscript has been accepted for publication in Development, pending our standard publication integrity checks.

© 2024. Published by The Company of Biologists under the terms of the Creative Commons Attribution License (https://creativecommons.org/licenses/by/4.0/).
